# The Influence of Single-Walled Carbon Nanotubes on the Aging Performance of Polymer-Modified Binders

**DOI:** 10.3390/ma16247534

**Published:** 2023-12-06

**Authors:** Svetlana Obukhova, Evgeniy Korolev, Vitaliy Gladkikh

**Affiliations:** 1 Department of Urban Planning, Institute of Architecture and Urban Planning, National Research Moscow State University of Civil Engineering, Moscow 129337, Russia; 2 Scientific and Educational Center “Nanomaterials and Nanotechnologies”, National Research Moscow State University of Civil Engineering, Moscow 129337, Russia; korolevev@nocnt.ru; 3 Research Center «MGSU Stroy-Test», National Research Moscow State University of Civil Engineering, Moscow 129337, Russia; gladkich_87@mail.ru

**Keywords:** single-walled carbon nanotubes, polymer-modified binder, aging, performance grade, rheology, multiple stress creep recovery, fatigue cracking, relaxation, durability

## Abstract

The use of polymer-modified binders in asphalt concrete makes it possible to increase the efficiency and durability of highways. However, at present, there is an important and unresolved problem in this area, making it impossible to fully exploit the potential of modified binders. This is a tendency of aging processes that leads to the premature destruction of the pavement. In many literary sources, it is reported that reasons are related to the peculiarity of the chemical composition and occur at the submicron level. Therefore, the influence of single-walled carbon nanotubes has been studied for a better understanding of aging processes. The aging processes of the RTFOT (rolling thin film oven test) and PAV (pressure aging vessel) modified with SBS (styrene–butadiene–styrene) polymer, single-walled carbon nanotubes, and waste industrial oil were simulated in a laboratory furnace. Microstructural features were studied using the method of infrared spectral analysis. The dependences of viscoelastic properties on the component composition of binders were investigated. The optimal content of single-walled carbon nanotubes (0.001%), SBS (styrene–butadiene–styrene) polymer (3.5%), and waste industrial oil (4%) in the binder composition was established, which synergistically improved the performance of the modified binder from PG (52-22) (performance grade) to PG (64-34). It was established that single-walled carbon nanotubes provide improvement in the durability parameter ∆*T*_c_ binder by 150%, improved relaxation properties at low temperatures, and resistance to fatigue damage.

## 1. Introduction

At present, a widely used group of materials in road construction is polymer–bitumen compositions, also known as polymer-modified binders (PMB) [1,2,3]. In general, it is a composition consisting of a rationally selected ratio of petroleum bitumen, styrene–butadiene–styrene polymer, and plasticizer, if necessary. Depending on the specified requirements for polymer-modified binders, they may contain surfactants, antioxidants, and nano- and micro-dimensional additives [4,5,6,7]. All these substances are aimed at improving the quality of the polymer-modified binder. But at the same time, they significantly complicate the system, making it less stable and, as a result, more susceptible to the processes of delamination and aging [8].

### 1.1. The Aging Process in Bitumen

The term “bitumen aging” in a generalized form combines the whole set of reversible and irreversible changes in its chemical composition, physical transformations, and changes in structural and mechanical parameters. These changes occur during the production of bitumen, its storage, transportation, technological processing, and operation, i.e., during the entire lifecycle of the bitumen binder. In modern studies, it is customary to separate short-term and long-term aging. The short-term aging process of bitumen takes place in a thin film of bitumen at high temperatures (150–200 °C). This short-term aging occurs when bitumen is connected to mineral material. Long-term or operational aging occurs at lower temperatures (less than 80 °C) but for a longer time during the operation of asphalt–concrete pavement. The short-term and long-term aging processes have different characteristics and differ in the rate of flow.

In the process of the short-term aging of bitumen, its group composition changes [9]. The number of oil fractions decreases, and the number of resinous–asphaltene fractions increases. These transformations occur because of oxidative reactions and the polymerization of light fractions, including their partial evaporation. It is possible to describe the ongoing processes in more detail using Semenov’s theory of chain reactions [10]. In the initial period of the short-term aging of bitumen, because of the interaction of hydrocarbons and oxygen in the air, peroxide and hyperoxide compounds are formed. These compounds are unstable, so they break down into free radicals. These trigger new chains of oxidative reactions. In the process of oxidative reactions, oxygen molecules are absorbed (embedded in chains), which leads to the destruction of high-molecular hydrocarbons (asphalt–resinous complexes). The oxidation reaction proceeds until the asphalt–resinous complexes turn into unsaturated chemical compounds, which are further polymerized, i.e., compacted, forming high-carbon compounds. Many studies [11,12,13,14] have reported that the short-term aging of bitumen leads to a natural change in its structure and properties. Viscosity, heat resistance, stiffness, and elasticity increase, but at the same time, there is a decrease in plasticity, which leads to an increase in fragility.

In the long-term aging of bitumen, in addition to chemical processes (oxidation) at low operating temperatures, physical processes also flow. These physical processes also change properties. These physical processes are associated with the formation of equilibrium supramolecular structures, leading to the hardening (solidification) of bitumen. There are several different theoretical descriptions of this process. Traxler and Coombs [15] reported that the physical aging of bitumen is associated with the manifestation of colloidal properties, i.e., the transition of bitumen from a sol structure to a gel structure. Gussfeldt [16] reported that the physical aging of bitumen during operation is associated with a change in the state of peptization of asphaltenes by maltenes. After that, a new adsorption equilibrium is established between the polar components of bitumen. The paraffin included in the bitumen crystallizes and, therefore, affects the hardening processes. In the process of spreading (remelting) bitumen, all these processes become reversible, but in the conditions of the operation process, these processes are irreversible.

### 1.2. The Aging Process in Polymer-Modified Binders

The term “aging of polymer-modified binder”, as well as the term “bitumen aging” in a generalized form, combines the whole set of reversible and irreversible changes in its chemical composition, physical transformations, and changes in structural and mechanical properties occurring during the production of polymer-modified binders, its storage/transportation, technological processing, and operation, i.e., during the entire lifecycle of the polymer-modified binder. Polymer-modified binders are complex multicomponent systems in which polymer and other additives, if available, make a significant contribution to the aging process. Therefore, despite numerous studies, at present, there is no reliable mechanism for the aging of polymer-modified binders. Due to the different nature and chemical properties of polymers, complex mechanisms of interaction occur between bitumen with polymers and modified bitumen with mineral aggregate [17]. It is worth noting that there are many studies focused on the study of aging on the structure and properties of polymer-modified binders [18,19,20,21,22,23,24,25,26,27,28,29].

Rheological methods are widely used to establish the thermal properties of polymer-modified binders [21,22]. Fourier transform infrared (FTIR) spectroscopy is also widely used to determine the polymer content in the modified binder and the effect of the thermo-oxidative aging process on the bitumen and SBS copolymers [23,24,25,26]. Fluorescence microscopy (FM) is used to assess the degree of dispersion of polymer, as well as its effect on the aging processes and morpho-structural features of the modified binder [27,28,29]. Therefore, the authors in [18] studied the elemental composition of polymer-modified binders (with several varieties of polymer) aged by the RTFOT method. They found that the content of the carbon component increased in all the aged samples studied, but the authors reported that they received mixed results in the presence of hydrogen, sulfur, and nitrogen. No clear trends were found for carbon, hydrogen, nitrogen, and sulfur due to polymer modification. Therefore, the authors also concluded that the aging process of polymer-modified binders largely depends on the type and level of modification [20]. The researchers in [19] found that there is not enough polymer in bitumen to form a strong polymer mesh, so it is necessary to introduce additional crosslinking additives. It was also established that an excess of polymer and crosslinking additives will not always improve the properties and resistance of modified bitumen to short-term aging.

Currently, the use of nanoscale modifiers (carbon nanomaterials) as a structuring component for polymer-modified binders is widespread [5,7,30]. Carbon nanotubes (CNTS), fullerenes, and graphene are most often used to modify polymer–bitumen composites [5,31,32]. The aspect ratio of the length to the diameter of the carbon nanotube can be unusually high, in the order of 1·10^7^:1 or more. This ratio is the reason that all the properties of carbon nanotubes are extremely anisotropic (i.e., they depend on direction) and can be varied directionally, and is of great interest since it opens the possibility of directional formation of the structure of the material [33,34,35,36]. They are also used because they have unique physical and mechanical characteristics for the directional structure formation of building composites. Their use makes it possible to significantly increase the strength and structural stability of modified binders [5], but despite all the advantages of using carbon nanomaterials, they are characterized by a feature that prevents industrial application, namely that despite the achievement of the nanoscale parameter of carbon nanotubes and distribution uniformity in the binder, they tend to aggregate over time. This feature minimizes the initial effect of nano-modification. Therefore, studying the influence of carbon nanotubes on the aging of polymer-modified binders is an urgent task, especially in terms of the effect of the long-term aging of polymer-modified binders.

The aging process of polymer-nanomodified binders is complicated by the presence of SBS polymer and carbon nanotubes. When the bitumen is joined with the SBS polymer, its elastomeric phase swells due to the absorption of the maltenes fractions (oil fractions) [37,38,39], which results in two phases: a bitumen matrix (bitumen phase) and a polymer phase. Earlier, the authors of [5], using electron microscopy to study asphalt–resin complexes with nanotubes, installed the physical barrier effect, which is conducted as follows: the introduction of carbon nanotubes into bitumen leads to an increase in the dispersion of asphalt–resin complexes, resulting in the formation of structural elements (physical barriers) that prevent the coagulation of asphalt–resin complexes. Based on this information, we assume that this effect (the obstruction of the coagulation of asphalt–resin complexes by carbon nanotubes) will contribute to the slowing of the long aging of polymer-nanomodified binders, which, as mentioned earlier, according to the ideas of Traxler and Gusfeldt [15,16], is based on physical processes associated with the formation of supramolecular structures resulting from the coagulation of asphaltenes and the subsequent transition of bitumen from a sol structure to a gel structure. Therefore, the aim of this research work is to study the influence of carbon nanotubes on the aging of polymer-modified binders and to add conclusions on how carbon nanotubes, together with SBS polymer and waste hydrocarbon plasticizer, affect long-term aging processes. This study can offer insight into the possible processes arising in polymer-nanomodified binders during aging on the scale of binders.

## 2. Materials and Methods

### 2.1. Raw Materials and Characterization

**Figure 1:**
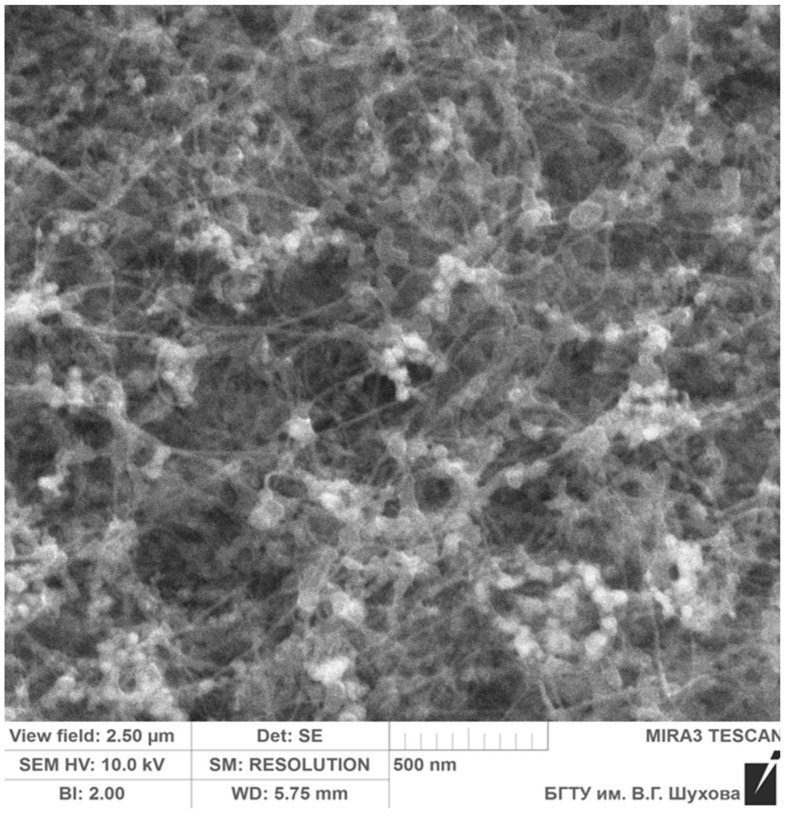
SEM image of the original single-walled carbon nanotubes.

Carbon nanotubes: single-walled carbon nanotubes (SWCNT) were obtained by the thermal evaporation of graphite in the presence of a Ni–Cr catalyst in an electric arc (Arc CNTs) containing graphite nanoparticles 20–100 nm long and metal nanoparticles 5 nm in diameter. Single-walled carbon nanotubes were synthesized at the Russian Academy of Sciences (Chernogolovka, Moscow, Russia) [40]. The image of the initial single-walled carbon nanotubes obtained using a scanning electron microscope is shown in Figure 1.

The initial single-walled carbon nanotubes are bundles of cylinders of various diameters of 2–100 nm and lengths up to 5–10 microns. The elemental chemical composition was studied using the scanning electron microscopy (SEM) method on a high-resolution scanning electron microscope (TESCAN MIRA 3 LMU, produced by TESCAN GROUP, Brno, Czech Republic). It was found that the initial single-walled carbon nanotubes contain 98.06% carbon (C) and 1.94% impurities (O, Al, Si, S, Cl, Fe, and Ni).

Plasticizer: waste industrial (minerals) oil-40A SN 300 brand, produced by LC Pushkinsky factory, Moscow, Russia. Waste industrial oil is a low-viscosity liquid, which is a complex mixture of paraffin, naphthenic, and aromatic hydrocarbons that meet the requirements of Russian State Standard 20799-88 [41]. The properties and requirements for the industrial oil are presented in Table 1.

Polymer: styrene–butadiene–styrene SBS L 30-01 A polymer. Thermoplastic linear butadiene–styrene polymer is a product of the block polymerization of styrene and butadiene in a solution of hydrocarbons in the presence of an organic lithium catalyst, powdered with calcium stearate or silicon dioxide. The characteristics of the polymer SBS 30L-01 correspond to the industrial standard IS 38.40327-98 [42], produced by the limited liability company Voronezhsintezkauchuk, Voronezh, Russia. The characteristics of the investigated polymer SBS 30L-01 are presented in Table 2.

Bitumen is an oil road bitumen of the PG 52-22, produced by LUKOIL-Nizhegorodnefteorgsintez LLC, Kstovo, Russia. Bitumen has been tested for compliance with the requirements of the Russian State Standard 58400.1-2019 [43]. The results of the laboratory tests of the physical and mechanical properties of bitumen PG 52-22 are shown in Table 3.

### 2.2. Methods for the Preparation of Polymer-Nanomodified Binder (PNMB)

**Figure 2:**
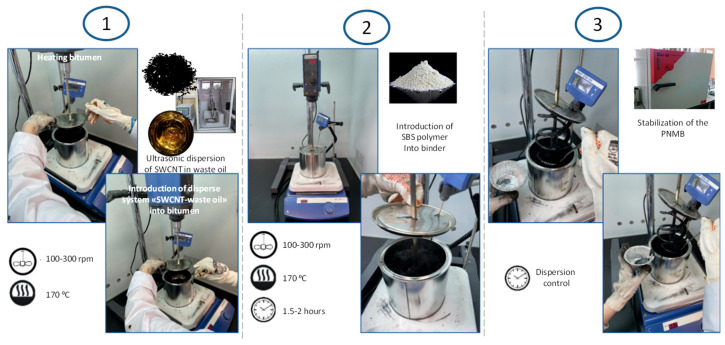
The technology of the preparation of polymer-nanomodified binder.

During several search studies, including loss control of nanomodified disperse systems “plasticizer–carbon nanotubes” during sample preparation, the use of various equipment for handling components, and various technological stages for linking components of the polymer-modified binder, were installed as the optimum preparation steps of polymer-nanomodified binders. These preparation steps provide stable results, as shown in Figure 2.

(1) In the first step, a nanomodified dispersed system, “waste industrial oil—single-walled carbon nanotubes”, is prepared using the submersible ultrasonic dispersant agent Vibra Cell VCX 750, produced by Sonics & Materials, Inc., Newtown, CT, USA. Ultrasonic dispersion continues until a nanoscale and distribution are achieved. It was previously established that the ultrasonic dispersion time depends on the percentage of carbon nanotubes and is in the range of 2–5 min [5]. Next, the bitumen is heated in a hermetic-lid container to a working temperature of 160–170 °C. Then, the IKA RW 20-bladed mixer and the heat sensor are immersed in the tank. The mixer is turned on at a speed of 100–300 rpm, and the dispersed system “waste industrial oil—single-walled carbon nanotubes” is slowly introduced for 2–3 min. Then, the system is mixed for 5–10 min.

(2) In the second step, at the end of the time, the SBS polymer is slowly introduced into the bitumen containing the nanomodified dispersed system for 3–10 min at a mixing speed of 100 rpm. At the end of the time, the mixer speed is increased to 300 rpm, and the container is hermetically closed with a lid. Then, a heat sensor is placed there. Mixing continues until the polymer is homogenized in the binder.

(3) In the third step, the polymer reaches homogenization, and the binder mixing ends. The polymer-nanomodified binder is placed in a drying cabinet and processed under a temperature of 135 °C for “ripening”. This will stabilize the structure of the polymer-nanomodified binder. The “ripening” stage lasts 1–2 h.

### 2.3. Characterization of Polymer-Nanomodified Binder

When constructing highways in the central part of the Russian Federation, the use of modified bitumen PG 64-34 is generally accepted. Therefore, in developing the optimal composition of the polymer-nanomodified binder, we sought to achieve this brand of bitumen. The binder formulations presented in Table 4 have been prepared for this purpose.

The final homogenization of the polymer was evaluated during the development of the optimal binder composition (Table 4). The presence of visible insoluble particles was determined with a glass stick. Samples No. 10, No. 11, and No. 12 failed the test and were not considered further. A rotational viscosity was checked for all samples of modified binders using a rotary viscometer. A rotational viscosity test of the binder was conducted to measure its resistance to flow for evaluating the workability. The rotational viscosities at 135 °C were determined using the Brookfield viscometer DV2TRV produced by AMETEK Brookfield, Middleboro, MA, USA. For 135 °C, 10.5 g of bitumen was taken and rotated with an *S*27 spindle. If the rotational viscosity was more than 3 Pa·s at a test temperature of 135 °C, then this sample was not used for further studies.

### 2.4. Study of the Short-Term Aging Process of Polymer-Nanomodified Binder: High-Temperature Characteristics

The characteristics of the PNMB were determined by the methods specified in accordance with AASHTO M 320 [44]:

1. The shear viscosity test of the binders was determined on a dynamic shear rheometer. The dynamic shear rheometer DSR (Anton Paar MCR 102 Smart Pave, produced by Anton Paar GmbH, Graz, Austria), based on the principle of adjustable shear deformation, was used to determine shear viscosity and to measure flow properties. The shear viscosity was determined using a geometry (two disks) with a gasket diameter of 25 mm (in accordance with AASHTO M 320 [44]).

2. Resistance to plastic deformation contributes to the resistance to the formation of plastic tracks (in summer and spring–summer periods). Resistance to plastic deformation will be established by determining shear resistance, which is equal to G*/sin *δ*. This is the ability of the modified binder to withstand the shear. Determined by the ratio of the complex shear modulus G* to the sine of the phase angle *δ*. Tests of the original bitumen and RTFOT aging bitumen will be carried out in accordance with the methodology set out in accordance with AASHTO T 315 [45].

3. To set the upper limit of the operating temperature range of the polymer-nanomodified binder (PG X), the maximum temperature at which the PNMB can retain the necessary properties will be set in accordance with the methodology set out in accordance with AASHTO R 29 [46].

4. To study the track-forming properties of polymer-nanomodified binders modified by SWCNT, a test of several multi-stress recovery creeps (MSCR) was conducted in accordance with the requirements of AASHTO T-350 [47]. The tests were conducted at two voltage levels of 0.1 kPa and 3.2 kPa. As a result of the test, the MSCR for non-recoverable compliance *J_nr_* (kPa^−1^) and the percentage of recovery *R* (%) of polymer-nanomodified binders were obtained to assess the resistance to track formation and elastic properties of the studied binders. A total of 10 cycles were used to determine the non-recoverable compliance and percentage of recovery. The indicators were determined at each loading cycle, and then averaging was performed. The non-recoverable compliance *J_nr_* (kPa^−1^) at every cycle was calculated using Equation (1):

(1) $$J_{nr}^{n}=\frac{\varepsilon_{r}^{n}-\varepsilon_{0}^{n}}{\tau_{0}},$$

where $\varepsilon_{r}^{n}$ is the strain value at the end of the recovery phase; $\varepsilon_{0}^{n}$ is the initial strain value at the beginning of the creep portion; and $\tau_{0}$ is the value of the stress level used in the loading cycle.

The percent recovery *R* at every cycle was calculated using Equation (2):

(2) $$R^{n}=\frac{\varepsilon_{c}^{n}-\varepsilon_{r}^{n}}{\varepsilon_{c}^{n}-\varepsilon_{0}^{n}\;},$$

where $\varepsilon_{c}^{n}$ is the strain value at the end of the creep portion.

### 2.5. Study of the Long-Term Aging Process of Polymer-Nanomodified Binder: Low-Temperature and Fatigue and Characteristics

Low-temperature and fatigue characteristics were determined for samples of polymer-nanomodified binders aged by the PAV (pressure aging vessel) method. PAV aging was determined using PAV 20-44000, produced by InfraTest LLC, Moscow, Russia. PAV aging is a method of aging under the influence of pressure and temperature and allows the simulation of the aging process during the period of operation in the road pavement from 5 to 10 years. Long-term aging was conducted in a chamber under a pressure of 2.1 MPa for 20 h at a temperature of 100 °C.

1. The low-temperature characteristics of the modified single-walled carbon nanotube PMB were determined on a beam-bending rheometer (BBR 20-44220, produced by InfraTest LLC, Moscow, Russia) at a temperature of −24 °C. The stiffness modulus (*S*) characterizes the resistance to constant loads, and the parameter *m* characterizes the rate of its change. The binder will provide resistance to low-temperature cracking if two conditions are met simultaneously: *S* = 300 MPa and *m* = 0.300 with a load duration of 60 s.

2. Fatigue cracking (cohesive cracking) of modified single-walled carbon nanotube PMB was determined on a dynamic shear rheometer (Anton Paar MCR 102 Smart Pave), according to Russian State Standard 58400.1-2019 [43] at a temperature of 19 °C. The measured values of the complex shear modulus G* and the phase angle *δ* characterize viscoelastic properties. Their multiplication value (G*·sin *δ*), according to Russian State Standard 58400.1-2019 [43] should be no more than 5000 kPa. In this case, the binder will exhibit viscoelastic properties and recover after load relief.

3. To establish the lower limit of the operating temperature range of the polymer-nanomodified binder (PG Y), we have identified the minimum temperature at which PNMB aged by the PAV method can retain the ability to relax stresses in accordance with the methodology set out in accordance with AASHTO R 29 [46].

For each percentage of polymer and single-walled carbon nanotubes, at least three samples were prepared and tested, and the standard deviation was no more than 2%.

### 2.6. Study of the Microstructural Changes in Polymer-Nanomodified Binder

Bitumen consists of aromatic, naphthenic, paraffin, and non-metallic derivatives containing sulfur, nitrogen, and metallic inclusions. Fourier Transform Infrared (FTIR) spectroscopy will allow the investigation of the nature of the interaction of components in the composition of polymer-modified binders before and after aging. Microstructural changes were determined using the IR Fourier spectrometer Agilent Cary 630, produced by Agilent Technologies, Santa Clara, CA, USA.

### 2.7. Study of the Relaxation Processes of Polymer-Nanomodified Binder

According to thermodynamic concepts, any system tends to move to its equilibrium state. Therefore, it is logical to assume that the physical changes occurring in polymer-modified binders during operation are associated with the transition of the structure to a thermodynamic state of equilibrium. Traditionally, the temperature of the asphalt–concrete pavement of highways is 150–160 °C. The cooling rate of the asphalt concrete up to 60 °C will be in the range of 0.5–1.5 °C. This speed is greater than that required to establish the equilibrium state of the structure in a highly viscous polymer-modified binder system at operating temperatures. Therefore, it takes time for the energy system to reach the most favorable equilibrium state, which is called relaxation time. Since the system is brought out of equilibrium by a change in external thermal energy, this kind of relaxation can be called thermal. During operation, the temperature of bitumen is constantly changing, which significantly complicates the flow of thermal relaxation processes, which, in turn, contributes to the formation of a structure that differs from the structure that was formed after the course of relaxation processes at a constant temperature. The consequence of the formation of equilibrium structures in polymer-nanomodified binders is their heterogenization, which in some cases may result in syneresis [48]. During thermal relaxation in bitumen, all properties change.

To characterize the thermal relaxation in polymer-nanomodified binders, we will use the durability parameter ∆Tc in this research work. This parameter was first proposed by Anderson [49] to study the loss of extensibility of aged bitumen binder, which characterizes the relationship between the properties of bitumen and cracks in the asphalt–concrete coating that are not associated with the load. This parameter ∆*T_c_* is proposed to be considered to be a criterion for assessing the loss-of-relaxation properties of bitumen. It is determined by the following equation [49]:

(3) $$∆T_{c}=T_{c(S)}-T_{c\left(m\right)},$$

where *T_c_*_(*s*)_ is isomodular temperature, °C; and *T_c_*_(*m*)_ is critical temperature, °C.

The isomodular temperature *T_c_*_(*s*)_ when the stiffness *S* is 300 MPa is calculated according to Equation (4):

(4) $$T_{c(S)}=T_{1}+\left[\frac{\log300-\log S_{2}}{\log S_{1}-\log S_{2}}\cdot \left(T_{1}-T_{2}\right)\right]-10,$$

where *T*_1_ is the lowest temperature at which both conditions are met: *S* ≤ 300 MPa and *m* ≥ 0.300 °C; *T*_2_ is the highest temperature at which one of the conditions is met: *S* ≤ 300 MPa or *m* ≥ 0.300 °C; *S*_1_ is stiffness determined within 60 s at temperature 1; *S*_2_ is stiffness, determined within 60 s at temperature 2; *m*_1_ is parameter *m* at temperature 1; and *m*_2_ is parameter *m* at temperature 2.

The critical temperature *T_c_*_(*m*)_, characterizing the ability of the binder to stress relaxation when the parameter *m* is 0.300, is as per Equation (5):

(5) $$T_{c(m)}=T_{1}+\left[\frac{\log300-\log S_{2}}{\log S_{1}-\log S_{2}}\cdot \left(T_{1}-T_{2}\right)\right]-10,$$

The durability parameter ∆*T_c_* allows us to quantify the loss-of-relaxation properties as the polymer-modified binder ages. The lower the value of the durability parameter ∆*T_c_*, the higher the tendency of the binder to age and, as a result, the deterioration of the relaxation ability of the binder.

## 3. Results and Discussion

Bitumen is a temperature-sensitive material whose viscosity is one of the most important parameters, which is a measure of its flow resistance affecting workability. The viscosity of basic and modified carbon nanotube PMB at a temperature of 135 °C was determined and shown in Table 5. When introducing carbon nanotubes, the problem is ensuring the uniformity of their distribution in the binder volume. Therefore, to determine the uniformity of the SWCNT distribution, the viscosity samples were taken from the upper and lower parts.

As seen in Table 5, the technology used for the preparation of PMB modified by nanotubes ensures the even distribution of the nanotubes in the binder volume. Therefore, the difference in the dynamic viscosity of samples taken from the upper and lower parts is no more than 1%. It was found that the addition of carbon nanotubes (PNMB compositions No. 2, No. 3, No. 5, No. 6, No. 8, and No. 9) increases the viscosity of the PMB [50]. The increase in viscosity is explained by a decrease in the penetrating ability of bitumen due to the structuring of the system with carbon nanotubes. This increase in viscosity affects the physical and rheological properties of the modified binder, making it stiffer. As the viscosity of the binder increases, a higher mixing temperature of the asphalt–concrete mixture in production is required. However, the entire viscosity of the polymer-modified binder modified by carbon nanotubes meets the requirements of the Superpave specifications (ASTM D6373) (i.e., 3000 cP) and is sufficiently fluid to be pumped during the operation of the asphalt mixing plant.

The influence of single-walled carbon nanotubes on the properties of developed polymer-modified binders is presented in Table 6.

### 3.1. Study of the Short-Term Aging Process of Polymer-Nanomodified Binder: High-Temperature Characteristics

**Figure 3:**
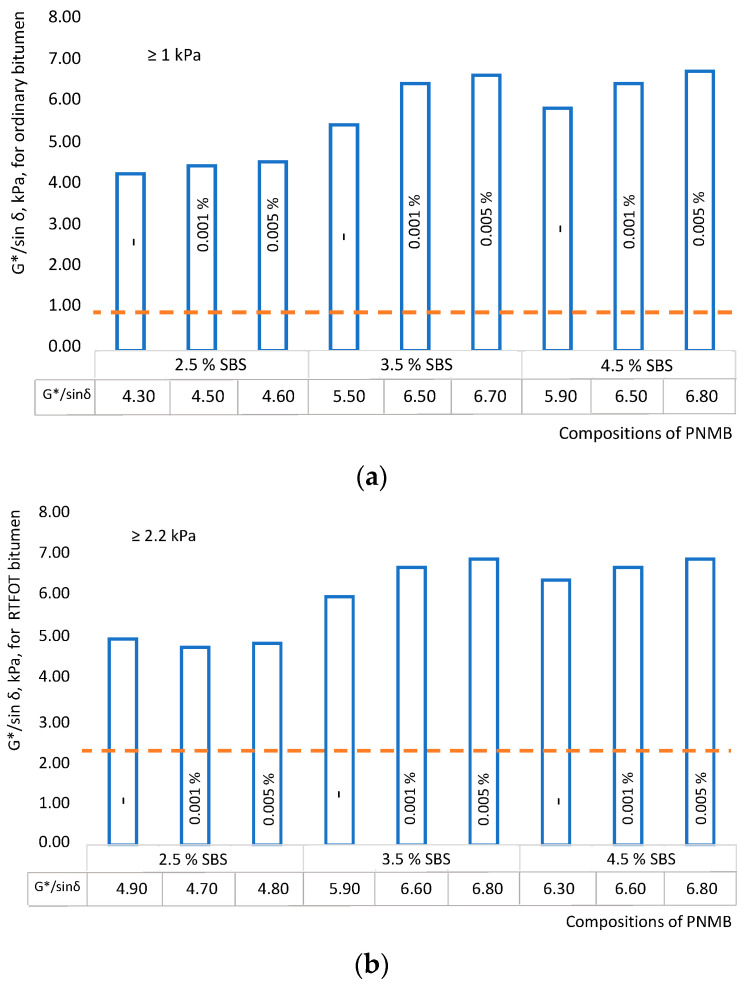
Influence of single-walled carbon nanotubes on the rutting factor (G */sin *δ*): (**a**) original modified binder; (**b**) modified binder aged by RTFOT.

**Figure 4:**
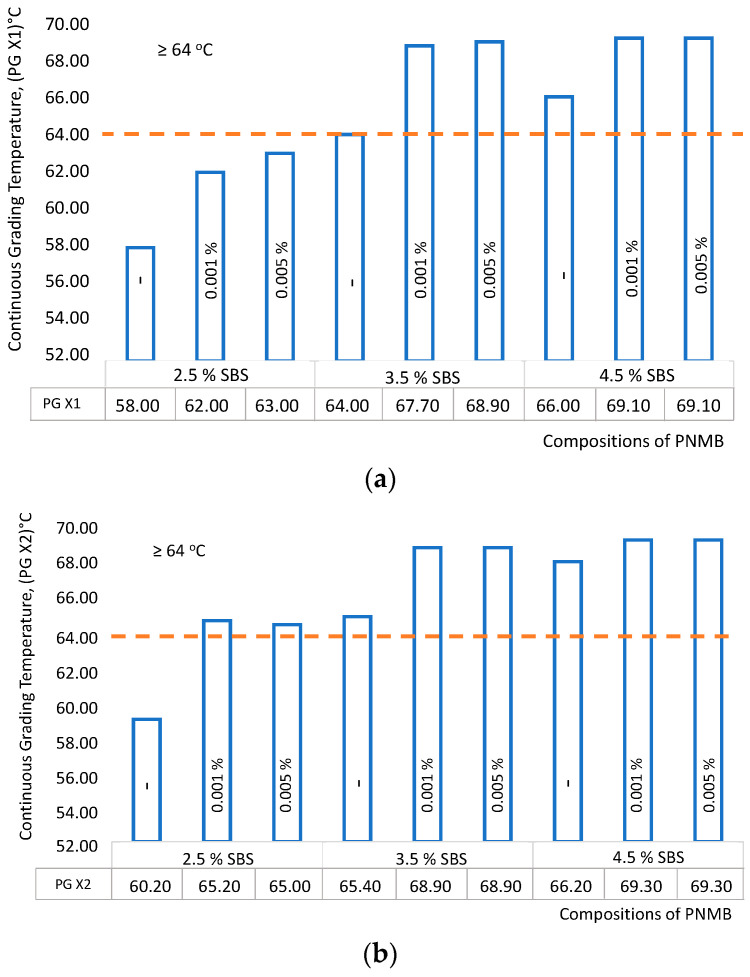
Influence of single-walled carbon nanotubes on the fracture temperature under high temperature: (**a**) original modified binder; (**b**) modified binder aged by RTFOT.

The rutting factor test was used to study the effect of single-walled carbon nanotubes on the characteristics of polymer-modified binders at high operational temperatures. The test results, i.e., the coefficients of formation of ruts and the corresponding fracture temperatures, are shown in Figure 3a and Figure 4a. The corresponding fracture temperatures are shown in Figure 3b and Figure 4b.

The value of G*/sin *δ* for non-aged binders should be at least 1.0 kPa and 2.2 kPa for binders to short-term aging according to the RTFOT method. Figure 3a shows that in PMB samples with a polymer content of 3.5%, the introduction of single-walled carbon nanotubes in an amount of 0.001–0.005% (compositions No. 5 and No. 6) allows the improvement of the rutting factor (G*/sin *δ*) up to 21%. Additionally, SWCNTs provide a stronger and more stable structure of the PNMB for the short-term aging process [51]. After aging by the RTFOT method, the rutting factor (G*/sin *δ*) for these samples did not change. The introduction of single-walled carbon nanotubes into PMB samples with a high polymer content of 4.5% (compositions No. 8 and No. 9) also makes it possible to improve the rutting factor (G*/sin *δ*), but in a smaller amount of 15% of the relative base composition (No. 7). It does not make significant changes in comparison with nanomodified samples (No. 5 and No. 6). Based on this, it can be concluded that when selecting the optimal composition of a nanomodified binder, a larger amount of polymer in combination with nanomodifiers will not always improve the properties of PMB more significantly. See, for example, the polymer-modified binder system with multi-walled carbon nanotubes [19].

In practical terms, the most promising samples are No. 5 and No. 6, which have demonstrated high sustainability in the formation of ruts at high temperatures (Figure 3). The performance of polymer-nanomodified binders at high temperatures has also improved; see Figure 4.

It was established that the performance characteristics of the modified binders at high temperatures increased from PG 52 to PG 64 with a polymer content of 3.5% and SWCNT 0.001–0.005% (i.e., PNMB No. 5 and No. 6), which once again confirms that SWCNT has a significant impact on performance improvement at high operating temperatures. Recall that for our research conditions, the samples of modified binders must meet the characteristics of the PG (X-Y)—PG 64-34. Unfortunately, PNMB samples No. 1, No. 2, and No. 3 do not meet the specified requirements.

For a more detailed study of the track-forming properties of polymer-modified binders modified by SWCNTs, the multiple stress creep recovery (MSCR) test was conducted. Table 7 summarizes the results of the MSCR test.

The MSCR test established a non-recoverable creep (*J_nr_*) and a recovery percentage (*R*) of polymer-nanomodified binders. It allows the assessment of the resistance to track formation and elastic properties of the studied binders. According to the results presented in Table 7, the introduction of single-walled carbon nanotubes (compositions No. 5, No. 6, No. 8, and No. 9) values of *J_nr_* (non-recoverable creep) both at a voltage level of 0.1 kPa and at 3.2 kPa gradually decreased. Regardless of the SWCNT content in the studied range of 0.001% (No. 5 and No. 8) and 0.005% (No. 6 and No. 9), these PNMB samples are characterized by the lowest *J_nr_* value, which indicates a significant improvement in resistance to track formation. The recovery percentage results show that PMBs modified by single-walled carbon nanotubes are restored more than the base composition of PMBs (No. 4 and No. 7) in the reduction cycles. The higher the recovery percentage, the higher the elasticity of the modified binders.

### 3.2. Study of the Long-Term Aging Process of Polymer-Nanomodified Binder: Low-Temperature and Fatigue and Characteristics

**Figure 5:**
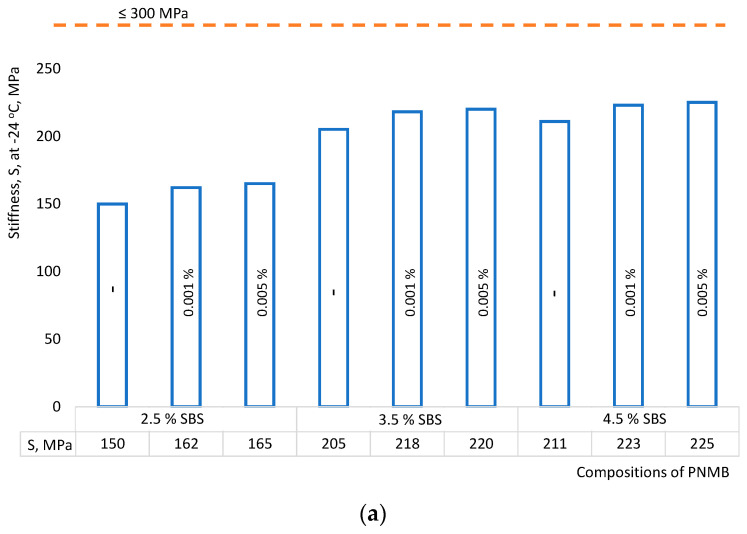

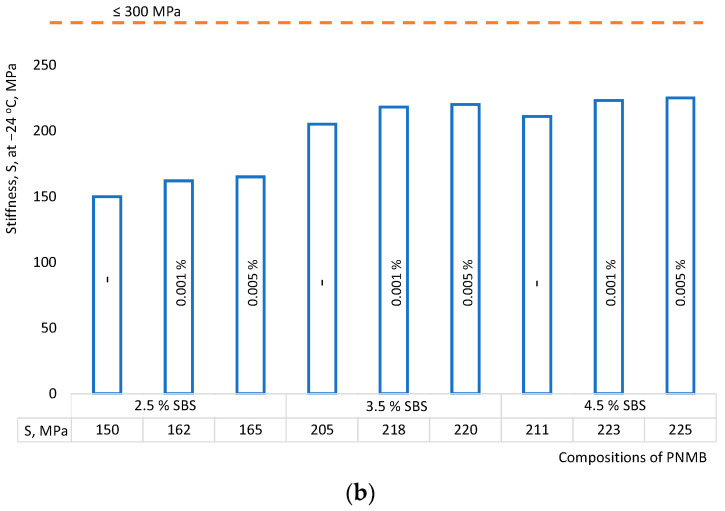
Influence of single-walled carbon nanotubes on: (**a**) stiffness *S*; (**b**) parameter *m* (creep) at low temperatures of the modified PAV aged binder.

The low-temperature characteristics of the PMB modified by single-walled carbon nanotubes were determined on a beam-bending rheometer (BBR) at a temperature of −24 °C. The measured values of stiffness *S* and parameter *m* are shown in Figure 5.

The bitumen binder will provide resistance to low-temperature cracking if two conditions are met simultaneously—*S* ≤ 300 MPa and *m* ≥ 0.300 with a load duration of 60 s. Figure 5a shows that the stiffness values of *S* increased slightly for all nanomodified binders. The average increase was no more than 7%. This allows us to conclude that single-walled carbon nanotubes in the studied range do not contribute to the deterioration of the low-temperature characteristics of the binder. The higher the *m* value, the greater the ability to diffuse the stress accumulated, indicating higher anti-cracking performance. The introduction of single-walled carbon nanotubes in PMB (No. 5 and No. 6) improves this parameter *m* by 10%, which leads to improved resistance to low-temperature cracking for these compositions. This is consistent with the data obtained for polymer-modified binder systems with multi-walled carbon nanotubes [50].

**Figure 6:**
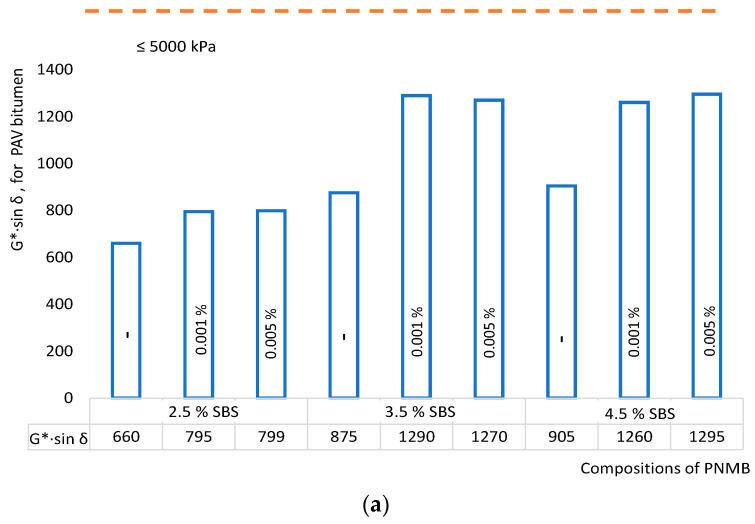

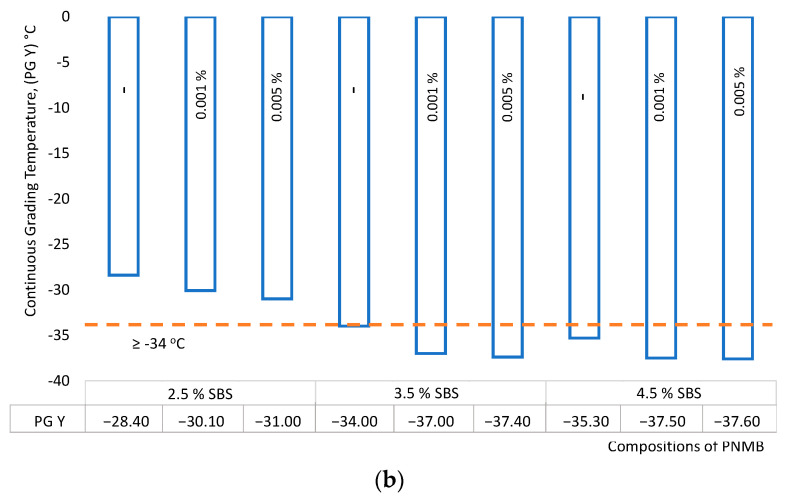
Influence of single-walled carbon nanotubes on (**a**) fatigue resistance; (**b**) temperatures of fatigue destruction on modified binder aged by the PAV method.

The fatigue characteristics of the PMB modified by single-walled carbon nanotubes were determined on a dynamic shear rheometer (DSR) at a temperature of 19 °C. Fatigue resistance G*·sin *δ* and temperatures of fatigue destruction are shown in Figure 6.

PMB modification by single-walled carbon nanotubes makes it possible to improve the fatigue resistance G*·sin *δ* (Figure 6a) up to 47% (No. 5). This indicates that after long-term aging (PAV), the nanomodified binder can effectively exhibit viscoelastic properties and recover after the load is removed. It characterizes nanomodified binders as systems with polymer mesh reinforced by single-walled carbon nanotubes that are resistant to long-term aging. The temperature performance of nanomodified binders has also improved from PG−22 to PG−34 with a polymer content of 3.5% and a SWCNT of 0.001–0.005% (i.e., PNMB No. 5 and No. 6), which further confirms that the SWCNT has a significant impact on improving performance at lower operating temperatures (Figure 6b).

Summarizing the results obtained on the development of PMB modified by single-walled carbon nanotubes, it can be concluded that the most promising and optimal compositions are PNMB No. 5 and No. 6.

### 3.3. Study of the Microstructural Changes in Polymer-Nanomodified Binder

**Figure 7:**
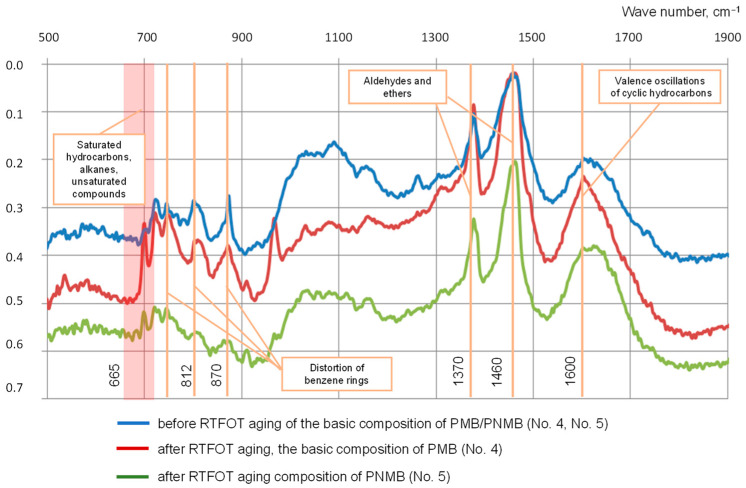
Influence of single-walled carbon nanotubes on the microstructural changes in polymer-nanomodified binder.

The method of infrared spectroscopy made it possible to study the effect of single-walled carbon nanotubes on the nature of the interaction of components in polymer-modified binders before and after aging. The results of the studies on samples No. 5 and No. 4 are shown in Figure 7.

Analyzing the results obtained, we can conclude the following: an increase in the absorption intensity of the peak of 1600 cm^−1^ indicates the ongoing processes of destructive thermo-oxidative aging, and in this case, the transition of resins to asphaltenes occurs. An increase in the absorption intensity of the peak of 1370 cm^−1^ indicates an increase in aldehydes and esters, which occurs because of thermo-oxidation processes in unsaturated compounds. A decrease in the absorption of deformation vibrations characteristic of aromatic compounds—triplet 747, 812, 870 cm^−1^—indicates a decrease in the content of light aromatic compounds. A decrease in the absorption of peaks 700–720 cm^−1^ indicates a decrease in the concentration of alkanes and aliphatic radicals, i.e., there is a decrease in the oil content in the binder after the thermo-oxidative aging process. A decrease in the absorption of peaks 665–700 cm^−1^ indicates the consumption of unsaturated compounds because of thermal oxidation processes. According to the data obtained (Figure 7), the compositions of PNMB No. 5 are characterized by a less intense decrease in peak absorption, which allows us to conclude that compositions with single-walled carbon nanotubes will be characterized by a more stable structure [5].

### 3.4. Study of the Relaxation Processes of Polymer-Nanomodified Binder

To study the influence of single-walled carbon nanotubes on the processes of operational aging, we studied relaxation processes occurring in binders. Isomodule temperature *T_c_*_(*s*)_, critical temperature *T_c_*_(*m*)_, and the durability parameter ∆*T_c_* were calculated for this purpose, which is presented in Table 8.

The durability parameter ∆*T_c_* quantifies the loss-of-relaxation properties as the long-term (PAV) aging of the polymer-modified binder. The lower the value of ∆*T_c_*, the binder is more susceptible to aging processes and loss of relaxation of emerging stresses and cracking. PMB modified by single-walled carbon nanotubes improved the durability parameter. The best synergistic effect in the “single-walled carbon nanotubes-SBS polymer” system is observed in sample No. 5. Therefore, 3.5% SBS polymer and 0.001% SWCNT provided an improvement in the durability parameter by 150%, which characterizes it as a polymer-nanomodified binder with higher relaxation properties at low temperatures. This confirms our assumption that single-walled carbon nanotubes preventing coagulation of asphalt–resin complexes contribute to slowing the operational aging of polymer-modified binders, which is based on the physical processes associated with the formation of supramolecular structures that occur due to the coagulation of asphaltenes.

A further increase in the content of SBS polymer and single-walled carbon nanotubes does not provide additional improvement in properties. Therefore, it can be concluded that the effect of introducing carbon nanotubes into the systems under study is limited.

## 4. Conclusions

In this study, a comprehensive evaluation of the influence of single-walled carbon nanotubes in combination with waste industrial oil and SBS polymer as a bitumen modifier in different amounts was investigated. Several laboratory tests were conducted, including physical, rheological, and relaxation tests. The conclusions obtained from the test results can be summarized as follows:

(1) The introduction of single-walled carbon nanotubes into a polymer-modified binder increased the viscosity of the system. The increase in viscosity is explained by a decrease in the penetrating ability of bitumen due to the structuring of the system with carbon nanotubes. However, the entire viscosity of the PMB modified by carbon nanotubes meets the requirements of Superpave specifications ≤ 3 Pa·s, which does not require technological changes in the preparation of asphalt–concrete mixture.
(2) The distribution of SWCNT is uniform. The dynamic viscosity of the samples taken from the upper and the lower parts differs by not more than 1%.
(3) The test results of PMB modified by single-walled carbon nanotubes with any amount of added modifier showed improved characteristics of track formation and fatigue resistance compared to basic PMB. However, the recommended amount of SWCNT is 0.001%, SBS polymer is 3.5%, and waste industrial oil is 4% by mass of bitumen to ensure fatigue resistance, which improves the performance of the modified binder from PG (52-22) to PG (64-34).
(4) According to infrared spectral analysis, the composition of PNMB No. 5 has less intensive changes in peak absorption, which leads to the conclusion that the introduction of single-walled carbon nanotubes in a polymer-modified binder will contribute to the formation of a more stable structure.
(5) It has been established that the use of single-walled carbon nanotubes slows the operational (long-term) aging process of polymer-modified binders and increases the durability parameter ∆*T_c_* by 150%.

However, additional research at a deeper level is required to fully understand the influence of single-walled carbon nanotubes on polymer-modified binders. In addition, future research should focus on increasing the sensitivity of PMB modified by single-walled carbon nanotubes to cracking at low temperatures in an extended temperature and time range. In addition, different types of bitumen should be used to assess the impact. Asphalt–concrete mix-related tests and field tests should be conducted to verify the actual performance and potential of industrial applications.

## Figures and Tables

**Table 1:** Physical and chemical properties of waste industrial oil-40A SN 300.

Properties	Requirements of Russian State Standard 20799-88	Actual Value
Kinematic viscosity at 40 °C, mm^2^/s	61–75	75
Acid number, mg KOH per 1 g of oil	<0.05	0.035
Ash content, %	<0.005	0.004
Mass fraction of sulfur in oils from sulfurous oils, %	<1.1	0.9
Density at 20 °C, kg/m^3^	<900	890
Flashpoint, °C	≥220	242
Stability against oxidation:		
increment of acid number of oxidized oil, mg KOH per 1 g of oil	<0.40	0.35
increment of resin, %	<3.0	2.6
Solvent content in selective cleaning oils	non	non

**Table 2:** Physical and chemical properties of SBS 30L-01.

Properties	Actual Value	Method
Toluene solution viscosity 5.23%/25 °C, cSt	14 ± 5	ASTM D 445
Volatile matter, %m	≤0.8	ASTM D 5668
Ash content, %m	≤0.3	ASTM D 5667
Content of bound styrene, %m,	30 ± 1.5	Internal method of supplier
Melt flow index, 200 °C/5 kg, g/10 min	<1	ASTM D 1238
Flashpoint, °C	≥220	237
Tensile strength, MPa	15	ASTM D 3182
Tensile stress at 300% elongation, MPa	2.7	ASTM D 3182
Relative elongation at break, %	700	ASTM D 3182

**Table 3:** Physical and chemical properties of bitumen PG 52-22.

Name of Parameter	Requirements of Russian State Standard 58400.1-2019	Actual Value
Originally bitumen		
Flashpoint, °C	≥230	249
Dynamic viscosity, test temperature 135 °C	≤3 Pa s	0.83
Dynamic shear: G*/sin *δ* at 10 rad/s, at test temperature 52 °C	≥1 kPa	2.03
Bitumen aging by RTFOT		
Dynamic shear: G*/sin *δ* at 10 rad/s, at test temperature 52 °C	≥2.2 kPa	2.93
Bitumen aging by PAV		
Aging temperature by PAV, °C	90	90
Fatigue resistance: G*·sin *δ* at 10 rad/s, at test temperature 19 °C	≤5000 kPa	546
Low-temperature stability: Stiffness, *S*, at −12 °C	≤300 MPa	154
Low-temperature stability: Creep, *m*, at −12 °C	≥0.300	0.311

**Table 4:** Compositions of polymer-modified/nanomodified binders.

NMPB Number	Components of Polymer-Nanomodified Binder, %				Homogeneity of PNMB
	Bitumen PG 52-16	Waste Industrial Oil	SBS 30L 01	SWCNT	
1	100.0	4.0	2.5	-	yes
2	100.0	4.0	2.5	0.001	yes
3	100.0	4.0	2.5	0.005	yes
4	100.0	4.0	3.5	-	yes
5	100.0	4.0	3.5	0.001	yes
6	100.0	4.0	3.5	0.005	yes
7	100.0	4.0	4.5	-	yes
8	100.0	4.0	4.5	0.001	yes
9	100.0	4.0	4.5	0.005	yes
10	100.0	4.0	5.5	-	no
11	100.0	4.0	5.5	0.001	no
12	100.0	4.0	5.5	0.005	no

**Table 5:** Rational viscosity (top/bottom) of polymer-nanomodified binders at 135 °C.

PNMB Number	Requirements of ASTM D6373	Actual Value
1	≤3 Pa s	0.99/-
2	≤3 Pa s	1.23/1.22
3	≤3 Pa s	1.27/1.27
4	≤3 Pa s	1.07/-
5	≤3 Pa s	1.57/1.57
6	≤3 Pa s	1.59/1.59
7	≤3 Pa s	1.09/-
8	≤3 Pa s	1.58/1.59
9	≤3 Pa s	1.59/1.60

**Table 6:** Operational properties of polymer-nanomodified bitumen.

Name of Parameter	Russian State Standard 58400.1-2019 (PG 64-34)	No. 1	No. 2	No. 3	No. 4	No. 5	No. 6	No. 7	No. 8	No. 9
Originally bitumen										
Dynamic shear: G*/sin *δ* at 10 rad/s, at test temperature 64 °C	≥1 kPa	4.3	4.5	4.6	5.5	6.5	6.7	5.9	6.5	6.8
Continuous Grading Temperature, (PG X1) °C	-	58	62	63	64	68.7	68.9	66	69.1	69.1
Bitumen aging by RTFOT										
Dynamic shear:										
G*/sin *δ* at 10 rad/s, at test temperature 64 °C	≥2.2 kPa	4.9	4.7	4.8	5.9	6.6	6.8	6.3	6.6	6.8
Continuous Grading Temperature, (PG X2) °C	-	60.2	65.2	65.0	65.4	68.9	68.9	68.2	69.3	69.3
Bitumen aging by PAV										
Aging temperature by PAV, °C	100	100	100	100	100	100	100	100	100	100
Fatigue resistance: G*·sin *δ* at 10 rad/s, at test temperature 19 °C	≤5000 kPa	660	795	799	875	1290	1270	905	1260	1295
Low-temperature stability: Stiffness, *S*, at −24 °C	≤300 MPa	150	162	165	205	218	220	211	223	225
Low-temperature stability: Creep, m value, at −24 °C	≥0.300	0.355	0.357	0.358	0.325	0.357	0.358	0.314	0.323	0.328
Continuous Grading Temperature, (PG Y) °C	-	−28.4	−30.1	−31	−34	−37	−37.4	−35.3	−37.5	−37.6

**Table 7:** Multiple stress creep recovery (MSCR) test results.

PNMB	*J_nr_* (kPa^−1^)			*R* (%)	
	0.1 kPa	3.2 kPa	*J_nr_* % Diff	0.1 kPa	3.2 kPa
No. 4	3.247	3.635	9.5	0.60	0.26
No. 5	1.289	1.388	9.90	2.90	0.90
No. 6	1.275	1.384	10.20	2.90	0.90
No. 7	2.738	2.914	7.40	1.30	0.45
No. 8	1.265	1.384	10.20	2.97	0.96
No. 9	1.265	1.384	10.20	2.97	0.96

**Table 8:** Influence of single-walled carbon nanotubes on the relaxation processes of polymer-modified binders.

NMPB Number	Parameters of Evaluation of Relaxation Processes of PNMB		
	*T_c_*_(*s*)_	*T_c_*_(*m*)_	∆*T_c_*
No. 1	−30.0	−28.4	−2.1
No. 2 *	−32.0	−30.2	−1.8
No. 3 **	−32.6	−31.0	−1.6
No. 4	−34.5	−34.0	−0.5
No. 5 *	−36.5	−37.0	0.5
No. 6 **	−36.9	−37.4	0.5
No. 7	−35.7	−35.3	−0.4
No. 8 *	37.0	−37.5	0.5
No. 9 **	37.1	−37.6	0.5

Note: *—with SWCN 0.001%; **—with SWCN 0.005%.

## Data Availability

Data are contained within the article.
